# Harnessing multimodal approaches for depression detection using large language models and facial expressions

**DOI:** 10.1038/s44184-024-00112-8

**Published:** 2024-12-23

**Authors:** Misha Sadeghi, Robert Richer, Bernhard Egger, Lena Schindler-Gmelch, Lydia Helene Rupp, Farnaz Rahimi, Matthias Berking, Bjoern M. Eskofier

**Affiliations:** 1 Machine Learning and Data Analytics Lab (MaD Lab), Department Artificial Intelligence in Biomedical Engineering (AIBE), Friedrich-Alexander-Universität Erlangen-Nürnberg (FAU), Erlangen, 91052 Germany; 2 Chair of Visual Computing (LGDV), Department of Computer Science, Friedrich-Alexander-Universität Erlangen-Nürnberg (FAU), Erlangen, 91058 Germany; 3 Chair of Clinical Psychology and Psychotherapy (KliPs), Friedrich-Alexander-Universität Erlangen-Nürnberg (FAU), Erlangen, 91052 Germany; 4 Translational Digital Health Group, Institute of AI for Health, Helmholtz Zentrum München - German Research Center for Environmental Health, Neuherberg, 85764 Germany

**Keywords:** Biomarkers, Health care, Medical research, Signs and symptoms, Biomedical engineering

## Abstract

Detecting depression is a critical component of mental health diagnosis, and accurate assessment is essential for effective treatment. This study introduces a novel, fully automated approach to predicting depression severity using the E-DAIC dataset. We employ Large Language Models (LLMs) to extract depression-related indicators from interview transcripts, utilizing the Patient Health Questionnaire-8 (PHQ-8) score to train the prediction model. Additionally, facial data extracted from video frames is integrated with textual data to create a multimodal model for depression severity prediction. We evaluate three approaches: text-based features, facial features, and a combination of both. Our findings show the best results are achieved by enhancing text data with speech quality assessment, with a mean absolute error of 2.85 and root mean square error of 4.02. This study underscores the potential of automated depression detection, showing text-only models as robust and effective while paving the way for multimodal analysis.

## Introduction

Depression, often termed a silent epidemic, impacts approximately 300 million individuals globally, profoundly affecting their thoughts, behaviors, emotions, and overall well-being^1^. It is a major public health challenge, with an estimated 5% of adults suffering from this condition^2,3^. Depression does not discriminate; it can affect anyone, regardless of background. People who have experienced abuse, severe losses, or other stressful events are more susceptible to developing depression. The consequences of untreated depression can be severe, leading to impaired functioning in daily life, strained relationships, and in the worst cases, suicide^2^. Despite the availability of effective psychotherapeutic and psychopharmacological treatments, many individuals do not receive adequate support. For instance, over 75% of people in low- and middle-income countries lack access to the care they need. Barriers to effective treatment include insufficient investment in mental health services, a lack of trained healthcare providers, and the social stigma associated with mental disorders^2,4^. As a consequence, a significant number of individuals affected by depression may never receive adequate diagnoses and therefore, treatment^2^. Diagnosing depression primarily relies on clinical interviews and questionnaires such as the Patient Health Questionnaire (PHQ)^5^. This process can be time-consuming and susceptible to confounding influences such as recall or rater biases, making false-positive or false-negative results a possibility^6^. One of the major hurdles in depression treatment is the subjective nature of assessment, which can result in inconsistent evaluations and potentially inaccurate diagnoses^6^. Thus, it is of paramount importance to improve the understanding, diagnosis, and treatment of depression to allow more effective and accessible clinical care.

Recent advancements in artificial intelligence (AI) have opened up new possibilities for tackling complex health challenges such as depression. Among these innovations, large language models (LLMs) like GPT-4o^7^ have showcased impressive capabilities in understanding and generating natural language. By leveraging these models, we can extract subtle linguistic and behavioral features indicative of depression from multimodal data sources, such as text, audio, and video, providing more objective and reliable assessments that overcome the drawbacks of conventional assessment approaches. Looking to the future, the potential applications of LLMs in mental health care extend beyond detection. Imagine an AI assistant capable of automatically and continuously monitoring an individual’s mental health by analyzing their written, verbal, or video interaction with clinical practitioners upon the patient’s consent. Such an assistant could provide early warnings if signs of depression are detected, prompting individuals to seek professional help sooner and increasing the chances of successful intervention. Moreover, such AI systems could offer personalized recommendations for self-help and psychotherapeutic/psychopharmacological treatment options. An AI assistant could suggest activities to foster behavioral activation, provide cognitive restructuring exercises, facilitate improved interpersonal relationships, and offer practical problem-solving strategies. By delivering such interventions through an accessible and personalized platform, AI could empower individuals to take proactive steps in managing their mental health, thereby complementing traditional therapeutic approaches.

In recent years, several studies have explored the use of AI and multimodal approaches for depression detection, laying a solid foundation for future advancements. Research on social media-based depression detection has been particularly prolific. Deshpande et al.^8^ utilized emotion AI and sentiment analysis to detect depression on Twitter^9^ by analyzing a curated list of depression-related words. Yazdavar et al.^10^ developed a semi-supervised model that incorporated word usage patterns and topical preferences to identify clinical depression symptoms from Twitter posts. Similarly, Trotzek et al.^11^ employed machine learning models, including Convolutional Neural Networks (CNNs) on word embeddings, to detect early depression symptoms from social media messages. Expanding to other platforms, Islam et al.^12^ analyzed user comments on Facebook^13^ using decision trees for emotional and linguistic features and support vector machines (SVMs) for temporal analysis. Orabi et al.^14^ proposed detecting depression from Twitter data through optimized word embeddings and deep learning models, including CNNs and recurrent neural networks (RNNs). Cacheda et al.^15^ focused on early depression detection methods using social media data, emphasizing linguistic and behavioral analysis. Tadesse et al.^16^ utilized Natural Language Processing (NLP) and machine learning to detect depression in Reddit posts^17^, relying on lexicons of words commonly used by individuals with depression. Burdisso et al.^18^ introduced a text classifier for early risk detection tasks such as depression detection using incremental classification and explainable AI. Moreover, Guo et al.^19^ demonstrated improved depression detection capabilities in resource-constrained settings by leveraging the strengths of pre-trained language models and topic modeling techniques. Pérez et al.^20^ developed a semantic pipeline to estimate depression severity from social media, using a multi-class classification approach to differentiate severity levels. Their method, which incorporates clinical symptoms from the BDI-II questionnaire^21^, achieves state-of-the-art results on Reddit benchmark^17^. Additionally, Nguyen et al.^22^ enhanced depression detection by grounding their models in the PHQ-9^23^ questionnaire’s symptoms, improving out-of-domain generalization on social media datasets. By integrating these clinically relevant constraints, they enhanced model interpretability and maintained competitive performance compared to standard BERT-based^24^ approaches. This demonstrates the potential of symptom-based modeling to improve AI applications in mental health diagnosis.

Besides social media, an invaluable dataset for AI research in depression detection is the distress analysis interview corpus Wizard-of-Oz (DAIC-WOZ)^25,26^. It comprises audiovisual recordings of 142 participants interacting with a human-controlled virtual agent, designed to diagnose psychological distress conditions such as anxiety, depression, and PTSD. The extended DAIC-WOZ dataset (E-DAIC) represents an expansion of the original DAIC-WOZ dataset (DAIC), featuring a larger cohort of 275 participants who underwent semi-clinical interviews with a virtual interviewer. The 20-minute interview sessions are converted into written transcripts and supplemented with annotations of acoustic and visual cues. The dataset ensures diverse representation and includes data from both human-controlled and autonomous AI interviews, along with clinical annotations like PTSD Checklist Civilian Version (PCL-C) and Patient Health Questionnaire-8 (PHQ-8) scores^25–27^. Several studies have utilized text, audio, video, or multimodal approaches to detect depression using the DAIC or E-DAIC datasets. For instance, Gong et al.^28^ developed a topic modeling approach that facilitated context-aware analysis of lengthy interviews in the DAIC dataset by extracting relevant topics. Williamson et al.^29^ discovered that analyzing an avatar’s speech patterns can be a powerful way to identify depression, highlighting how the condition affects communication.

Other studies have integrated multiple modalities for enhanced depression detection. Nasir et al.^30^ explored multimodal features for depression classification, and found that i-vector (identity vector) modeling, a feature extraction technique, performed exceptionally well in audio analysis. Al Hanai et al.^31^ used long short-term memory (LSTM) networks to detect depression using both audio and text modalities. Stepanov et al.^32^ took a multimodal approach, fusing speech, language, and visual cues to predict depression severity, as measured by PHQ-8 scores, using the DAIC dataset. Fan et al.^33^ utilized a multi-scale temporal dilated CNN for depression detection, incorporating both text and audio features. Yin et al.^34^ predicted depression severity using a multimodal approach with bidirectional LSTM networks. Shen et al.^35^ identified two key predictors of depression severity in the DAIC dataset: spectral features extracted from audio recordings and behavioral cues extracted from interview transcripts, both of which proved to be strong indicators of depression severity. Prabhu et al.^36^ developed a multimodal depression detection system combining facial expressions (CNN-LSTM), text (LSTM), and audio (LSTM) features, leveraging transfer learning and ensemble techniques for enhanced performance. The E-DAIC dataset^27^ has been utilized in various studies as well. For instance, Makiuchi et al.^37^ proposed a multimodal approach for depression detection, combining text, audio, and facial features using deep learning techniques, including VGG-16^38^ for speech, BERT for language, and ResNet-50^39^ for visual features. Ray et al.^40^ introduced a multi-level attention-based network for predicting depression severity, highlighting the importance of textual information.

**Fig. 1:**
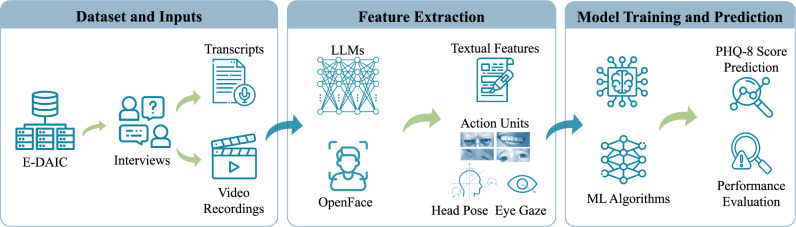
Graphical abstract of the proposed framework. (1) E-DAIC dataset including transcripts and video recordings^25^. (2) Feature extraction with Large Language Models (LLMs) and OpenFace^42^. (3) Model training for PHQ-8 score prediction and performance evaluation.

A significant limitation of prior research on text-based depression detection using the DAIC or E-DAIC dataset is the reliance on manual text preprocessing, feature extraction, and topic identification. This labor-intensive approach underscores the need for more automated and comprehensive methods. The current paper addresses these limitations by presenting a novel, fully automated approach built on the E-DAIC dataset, which leverages state-of-the-art LLMs to automate the extraction of depression-relevant features from interview transcripts. This automation enables the efficient identification of language features pertinent to depression, eliminating the need for manual processing. In our previous work^41^, we demonstrated the power of LLMs in uncovering valuable insights from textual data, leveraging the E-DAIC dataset to explore the potential of automated feature extraction. Our study revealed that LLMs can enhance the efficiency and accuracy of depression screening and diagnosis, by automating the analysis process and minimizing the need for manual review. Building upon this foundation, the current paper presents a novel approach that refines and expands our previous work. We introduce alternative prompts to improve the extraction of depression-related features from interview transcripts using state-of-the-art LLMs. This refinement enables more accurate and nuanced feature extraction, enhancing the automated analysis process. We then use these extracted features to build a machine-learning model that predicts PHQ-8 scores of individuals as a measure of depression severity. Furthermore, we explore the incorporation of visual cues extracted from video frames, including facial expressions, eye gaze, and head pose, using bidirectional LSTM networks. By fusing both textual and visual modalities, we construct a multimodal model for predicting depression symptoms. Our approach enables a comprehensive evaluation of the effectiveness of each modality, allowing us to identify which data modality is more effective in detecting depression symptoms. We evaluate the performance of each model using standard metrics to compare their effectiveness and determine the relative strengths of textual and visual features in detecting depression symptoms. Figure 1 provides a summary of our proposed approach.

The structure of the paper is outlined as follows: Section “Methods” provides a detailed description of the proposed method, while Section “Results and Discussion” presents and discusses the experimental findings.

## Methods

In this section, we describe our proposed method for depression detection. First, we detail the dataset used. Then, we consider the automated depression assessment based on textual data, including the feature extraction and training process. Following this, we explain a speech quality assessment that we suggest to potentially improve prediction accuracy. In Section “Visual features for automated depression assessment”, we outline our method for depression detection based on visual features. Finally, in Section “Multimodal features for automated depression assessment”, we present the proposed multimodal approach, which combines both textual and visual features for depression detection.

### Dataset description

Expanding upon DAIC, the E-DAIC dataset offers a collection of semi-clinical interviews facilitated by Ellie, a virtual interviewer, with accompanying transcripts and annotations of acoustic and visual cues. The virtual interviewer can be controlled either by a human in a Wizard-of-Oz setting or autonomously by AI, allowing for realistic simulation of clinical interviews. The dataset consists of 275 interview sessions, featuring a participant pool of 170 males and 105 females, which are then divided into three subsets: a train set of 163 instances, a development set of 56 instances, and a test set of 56 instances. The test set is solely constituted from the data collected by the autonomous AI. The dataset was carefully curated to ensure diverse speaker representation, with deliberate attention paid to age and gender distribution, resulting in a dataset reflecting the broader population’s diversity. Details regarding the size of each partition and speaker distribution over the partitions are given in Table 1. The provided visual features have been extracted using the OpenFace software^42^, and acoustic features have been extracted using the openSMILE tool^43^. The dataset also includes automatic transcription of the interactions using Google Cloud’s speech recognition service, participants’ audio files, as well as PTSD and PHQ-8 scores. The PTSD score ranges from 0 to 85, while the PHQ-8 score ranges from 0 to 24, with higher scores indicating greater depression severity^25–27^. In the provided development set, PHQ-8 scores range from 0 to 20, with PTSD severity scores ranging from 17 to 72. The train set exhibits PHQ-8 scores ranging from 0 to 23, with PTSD severity scores spanning from 17 to 85. Finally, in the test set, PHQ-8 scores range from 0 to 22, while PTSD severity scores range from 19 to 77. Figure 3 shows the distribution of PHQ-8 scores in each set of the dataset. As observed in the plots, higher PHQ-8 scores are rare, and the mean score in each set is below 10. This uneven distribution poses a challenge for machine learning models trained on this data. Since the model is exposed to fewer high PHQ-8 scores during training, it struggles to accurately predict the higher scores due to insufficient high-score examples.

**Table 1:** Number of participants and duration of the interviews included in the E-DAIC dataset^25^

Partition	# Participants	Duration [h:min:s]
Train	163	43:30:20
Development	56	14:47:31
Test	56	14:52:42
All	275	73:10:33

It is important to note that the E-DAIC dataset used in this study contains no protected health information (PHI). The dataset curators removed all identifying details, such as names, dates, and locations, from the audio recordings and transcriptions. Additionally, the facial features in the dataset are not detailed enough to identify individuals. The dataset is publicly available, and interested researchers can apply to receive access at https://dcapswoz.ict.usc.edu/. Research investigators planning to use similar methods on other datasets should be aware that they may encounter PHI and take necessary measures to ensure compliance with relevant regulations.

### Textual features for automated depression assessment

**Fig. 2:**
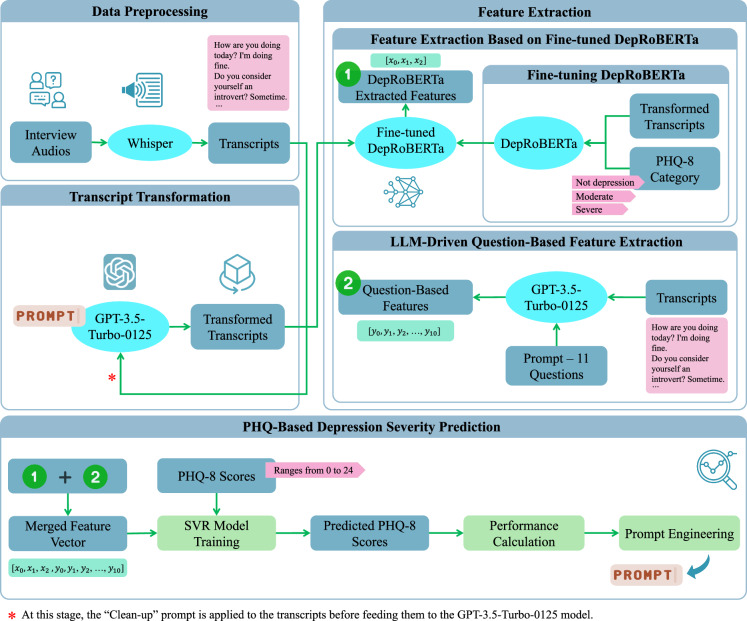
Overview of the proposed framework for depression detection based on textual data. (1) Data preprocessing using Whisper’s automatic speech recognition^44^. (2) Transcript transformation via GPT-3.5-Turbo-0125^47^. (3) Feature extraction by DepRoBERTa^49^ and an LLM-driven question-based method. (4) PHQ-based depression severity prediction: model training, PHQ-8 score forecasting, performance evaluation, and prompt engineering.

This section explores textual features for automated depression assessment. Based on the pipeline proposed in our previous work^41^, we extend our approach to improve the assessment of depression from interview transcripts. The proposed pipeline is depicted in Fig. 2. We begin by converting interview audio recordings into text using automatic speech recognition, followed by the application of LLMs to transform the transcripts and extract features pertinent to depression. Finally, we train the model using PHQ-8 scores as the target variable. Through iterative performance evaluations and prompt engineering, we refine the prompts utilized in the pipeline. Each component of the pipeline will be thoroughly explained in the following sections.

The E-DAIC dataset includes automated transcripts generated by speech-to-text systems, which are often incomplete and inaccurate, resulting in the loss of crucial context and details. Through inspection of several transcripts and by listening to the corresponding interview audios, we observed that significant questions and responses are sometimes reduced to simple ‘yes’ or ‘no’ answers, with the original question missing. Occasionally, the question is included without the corresponding answer. Furthermore, the conversational flow is frequently disrupted, with key phrases fragmented or essential background information missing. To address these limitations, we utilized OpenAI’s Whisper^44^ automatic speech recognition system to generate high-quality transcripts from raw interview recordings. The Whisper ‘large’ model stands out due to its unique robustness properties and superior performance across various datasets. While it achieves a word error rate (WER) of 2.7 on the LibriSpeech test-clean dataset^45^, its zero-shot capabilities allow it to outperform all benchmarked LibriSpeech models by significant margins on other datasets. Even the smallest zero-shot Whisper model is competitive with the best-supervised models when evaluated outside of the LibriSpeech test-clean framework. The best zero-shot Whisper models not only closely match human accuracy and robustness but also deliver a 55.2% average relative error reduction on diverse speech recognition datasets^44^. By employing the Whisper ‘large’ model and reviewing several transcripts, we observed an improvement in quality, with more context and information retained in the Whisper-generated transcripts. This analysis builds on our previous study, where we proposed this method to enhance the quality of E-DAIC dataset transcripts^41^.

Despite Whisper’s advantages, our examination of several transcripts revealed a few issues. During this inspection, we occasionally found that some answers to questions were missing. Upon reviewing the interview audio, we determined that this missing information was primarily due to low audio quality, which rendered certain parts unrecognizable. Additionally, we observed instances of word duplication in Whisper-generated transcripts, such as repeated phrases like “That’s not true. That’s not true. That’s not true...”. Similar duplication issues have also been noted by other researchers^46^. To remain consistent with our automated approach, we opted not to manually correct these duplications. Instead, we relied on LLMs in the subsequent transcript transformation step to help address these challenges by extracting the most significant topics related to depression.

To prepare the transcripts for depression detection, we refine them to make them clearer and more concise using a Clean-up Prompt: *"This interview involves a conversation with someone. Could you modify it by removing questions that don’t have an answer? Keep in mind that responses such as ‘yes’ and ‘no’ are also acceptable.”* As mentioned earlier, during our screening of Whisper-generated transcripts, we noticed some questions were stated without a corresponding answer. To address this, we developed this Clean-up Prompt as an automated solution. Our aim was to investigate whether removing questions that lacked answers could improve the performance of our proposed depression detection model, as questions without answers might confuse LLMs during the transcript transformation step. Next, we use three specific prompts to extract crucial information related to depression from the revised transcripts. These prompts help us identify key points and summarize relevant information. The three prompts used in our analysis are:

- Prompt 1 (derived from our previous study^41^): “*Your task is to read the following text which is an interview with a person and to summarize the key points that might be related to the depression of the person.”*
- Prompt 2 (refined from our previous study^41^): “*Your task is to read the following text which is an interview with a person and to summarize the key points that might be related to the depression of the person. Please be concise and write your response from the first-person perspective, as if you are the interviewee narrating about your own experiences.”*
- Prompt 3 (newly designed): “*Could you provide a summary of the main points concerning the mental health of the interviewee from the interview?”*

We applied these prompts to the revised transcripts using GPT-3.5-Turbo-0125, a state-of-the-art LLM developed by OpenAI^47^. Through prompt engineering, we iteratively designed and refined the prompts. We also utilized the GPT model to suggest alternative prompts, and by evaluating the whole pipeline and validating the model’s predictions using error metrics, specifically mean absolute error (MAE) on the test set, we selected the three most effective prompts that are presented in this paper. Additionally, we conducted a separate experiment where we bypassed the revision step and directly applied the three mentioned prompts to the original Whisper-generated transcripts, without applying the Clean-up Prompt. This allowed us to compare the performance of the prompts on both refined and raw transcripts.

Following the transformation and summarization of the interview transcripts, we utilize a pre-trained language model to examine the processed transcripts, as outlined in our previous research^41^. Specifically, we employ a fine-tuned RoBERTa^48^ language model, known as DepRoBERTa^49^, which is specifically designed for depression detection. This model is built upon RoBERTa-large^48^ and was pre-trained on Reddit^17^ posts related to depression. The ‘deproberta-large-depression’ model has shown exceptional performance in detecting depression levels in English social media posts^49^. Notably, DepRoBERTa emerged as the top solution in the Shared Task on Detecting Signs of Depression from Social Media Text at LT-EDI-ACL2022 and is available on the Hugging Face model hub^50^. The model can detect three different levels of depression: ‘not depression’, ‘moderate’, and ‘severe’ based on text data^49^.

**Fig. 3:**
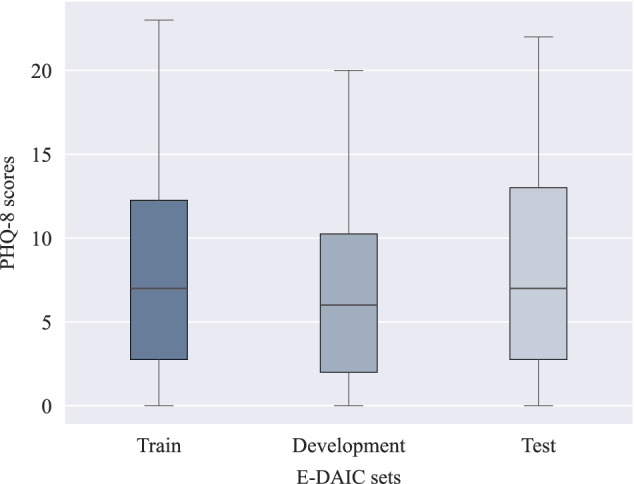
PHQ-8 score distribution in the E-DAIC^25^ dataset. The box plots show the median (horizontal line within each box), the interquartile range (IQR; represented by the edges of the box), and whiskers extending to 1.5× IQR from the box edges. The *y*-axis shows PHQ-8 scores ranging from 0 to 24.

To tailor the model to our dataset, we conducted fine-tuning of the model with a low learning rate of 5 × 10^−6^. As the DepRoBERTa model was initially trained on a dataset with three classes, we categorized the transformed transcripts into three labels based on their corresponding PHQ-8 scores to match the original model’s training data. The standard PHQ-8 score categories for depression diagnosis include mild symptoms (5–9), moderate symptoms (10–14), moderately severe symptoms (15–19), and severe symptoms (20–24), with scores below 5 indicating no depression^5^. Given the imbalanced distribution of PHQ-8 scores in the E-DAIC dataset, as illustrated in Fig. 3, particularly the scarcity of instances in the moderately severe and severe groups, we devised a simplified categorization scheme to address this imbalance. Specifically, scores of 14 or higher were categorized as ‘severe’, scores between 7 and 13 (inclusive) were labeled as ‘moderate’, and scores lower than 7 were designated as ‘not depression’. This scheme was designed to ensure the fine-tuned DepRoBERTa model had a sufficient number of instances in each category, thereby improving the model’s ability to learn effectively from the data. This categorization was not intended for clinical diagnosis but was crucial for balancing the dataset and avoiding the issue of the model being skewed by the scarcity of severe depression instances in the original E-DAIC dataset. Subsequently, we trained the model on the labeled data, evaluating its performance on the development set and implementing early stopping to prevent overfitting. This mechanism monitored the development loss at the end of each epoch. Specifically, we tracked the best development loss observed, which was computed using the cross-entropy loss function, and terminated the training if the development loss did not improve for three consecutive epochs. Following fine-tuning, we used the fine-tuned DepRoBERTa model to perform inference on the transformed transcripts. Each transformed transcript was provided as input to the model, which then produced an output representing the probabilities of the text belonging to each of the three depression classes: ‘not depression’, ‘moderate’, and ‘severe’. These probabilities, ranging between 0 and 1, served as features extracted from the model. For example, when the text “I am feeling very well.” was input into the model, the resulting output might be an array such as [0.966, 0.026, 0.008], corresponding to the probabilities for ‘not depression’, ‘moderate’, and ‘severe’ classes, respectively. This output indicates a high probability of ‘not depression’ and very low probabilities for the other two classes.

To further enrich our feature vector beyond what the DepRoBERTa model generated, we explored an additional approach to extract relevant information from the transcripts. Our aim was to develop a more nuanced and targeted set of features tailored to the unique characteristics of the interviews. To achieve this, we used the GPT-3.5-Turbo-0125 model. We provided the model with a selection of sample interviews from the dataset and tasked it with designing questions that could differentiate responses from individuals with and without depression. The model generated a set of questions based on the provided transcripts. We then used these questions to extract features based on each interview transcript. The 11 questions listed below were crafted to probe various aspects of depression, including emotional and physical well-being, mood changes, sleep disturbances, concentration difficulties, and past diagnoses.

1. Have you felt emotionally and physically well lately?
2. Have you noticed significant changes in your mood, such as feeling persistently sad, empty, or hopeless?
3. Have you experienced difficulties with your sleep, such as trouble falling asleep, staying asleep, or waking up too early?
4. Are you finding it challenging to concentrate on tasks or make decisions?
5. Have you lost interest or pleasure in activities you used to enjoy?
6. Have you ever been diagnosed with depression or experienced prolonged periods of feeling down or hopeless in the past?
7. Have you ever been diagnosed with PTSD (Post-Traumatic Stress Disorder)?
8. Have you been experiencing any financial problems recently?
9. Do you find it challenging to socialize and prefer solitary activities, indicating introverted tendencies?
10. Have you had thoughts of death or suicide, or have you made any suicide attempts?
11. Have you ever served in the military?

Next, we crafted a custom prompt and posed the questions to the GPT model, asking it to respond with one of the following answers: ‘YES’, ‘NO’, ‘To Some Extent’, or ‘Not Mentioned’. The prompt was formulated as follows: “Can you answer these questions from this text, which is an interview with a person, only with ‘YES’ or ‘NO’ or ‘To Some Extent’? If the question or corresponding answer is not found, answer ‘Not Mentioned’.” The model’s responses were then converted into a numerical feature vector, where ‘YES’ was mapped to 1, ‘NO’ to 0, and ‘To Some Extent’ to 0.5. In cases where the model responded with ‘Not Mentioned’, we initially assigned a value of NaN (Not a Number) and then substituted the mean value of the respective question within each of the train, development, and test sets separately.

In the subsequent step, we trained a support vector regression (SVR) machine learning model, using the PHQ-8 scores as the outcome variable. The model leverages the extracted features to predict PHQ-8 scores for each interview transcript. From the two feature extraction steps described above, we obtained a 14-dimensional feature vector for each interview: 3 features are derived from the fine-tuned DepRoBERTa model while 11 features are from the LLM-driven question-based feature extraction method. The SVR model uses a linear kernel, with the hyperparameter *C* = 1.0. We trained the SVR model exclusively on the train set and evaluated its performance on the untouched development and test sets, ensuring direct comparability with existing studies that use the same dataset configuration.

Additionally, to evaluate the impact of transcript transformation on depression detection, we conducted an additional experiment where we bypassed the transformation step entirely. In this analysis, we extracted features directly from the raw interview transcripts generated by Whisper using the fine-tuned DepRoBERTa model. We assessed two configurations: first, using DepRoBERTa features in conjunction with features derived from the question-based method, and second, using only the DepRoBERTa features without question-based features. In both cases, the extracted features were used to train the SVR model for predicting the PHQ-8 scores. The goal was to determine whether using raw transcripts without transformation could achieve comparable performance.

To further validate the robustness of our model and ensure its generalizability, we also performed a nested cross-validation analysis on the combined train and development sets. This additional evaluation involved an outer fivefold cross-validation loop, coupled with an inner fivefold cross-validation loop for hyperparameter tuning. Specifically, the inner loop utilized GridSearchCV from the Scikit-learn library^51^ to optimize parameters such as the kernel type, regularization parameter (*C*), and Kernel coefficient (*gamma*) for non-linear kernels. By leveraging GridSearchCV, we explored different hyperparameter combinations to identify the best model configuration. This nested cross-validation approach allowed us to assess the model’s performance more rigorously by leveraging multiple folds within the training data for both training and validation, while still keeping the test set untouched for final evaluation. The results of these evaluations are detailed in Section “Outcomes of Depression evaluation using textual data”.

The E-DAIC dataset poses a significant challenge due to the varying audio quality of the interview recordings. Background noise, inconsistent loudness levels, and other imperfections can degrade the accuracy of automated speech recognition (ASR) systems, resulting in incomplete or inaccurate transcripts and undermining the reliability of subsequent analyses. Even with advanced ASR systems like Whisper, which were shown to outperform conventional methods, limitations remain. To tackle this issue, we introduce an approach that aims to enhance the accuracy of our method. For automatic speech quality assessment, we used the Python Package NISQA^52,53^, which yields multidimensional audio quality predictions, including overall speech quality as well as quality dimensions such as noisiness, coloration, discontinuity, and loudness^52^. To ensure the reliability of our analysis, we applied the NISQA tool to all interview audios in the train, development, and test sets. We set a threshold for acceptable speech quality at 2.5, specifically for the overall speech quality score provided by NISQA, which ranges from 1 to 5^54^. This decision was made based on empirical observations and testing of the dataset. We found that an overall speech quality score of 2.5 or higher allowed us to include a sufficient number of interviews while still maintaining a standard for acceptable audio fidelity. The overall speech quality scores ranged as follows: in the train set, the scores ranged from a minimum of 1.34 to a maximum of 3.99; in the development set, from a minimum of 1.15 to a maximum of 4.09; and in the test set, from a minimum of 1.23 to a maximum of 3.48. After the quality check, 49 interviews from the train set (30.1%), 21 from the development set (37.5%), and 31 from the test set (55.4%) failed to meet the quality threshold. The results based solely on data that match the speech quality requirement are reported in the “Results” section.

### Visual features for automated depression assessment

As previously mentioned, the E-DAIC dataset comprises audiovisual recordings of semi-clinical interviews. Even though the publicly available version of the E-DAIC dataset does not contain the original video files, it provides visual features per video frame. The visual features that were extracted using the OpenFace software^42^ can be categorized into the following groups:

- Action Units (AU): A subset of 18 AUs, along with their presence and intensity. The Facial Action Coding System (FACS)^55^ is a system to taxonomize facial expressions by coding the movements of facial muscle groups into AUs. For instance, the activation of AU6 corresponds to the raising of the cheeks.
- Head Pose: The three-dimensional position of the head relative to the camera, as well as rotational data encompassing roll (rotation around the head’s front-to-back axis), pitch (rotation around the head’s side-to-side axis), and yaw (rotation around the head’s vertical axis)^56,57^.
- Eye-Gaze: The angle of the left and right eye gaze in radians^58^.

The E-DAIC dataset comprises a range of facial features, totaling 49, including head pose, eye gaze, and AUs. For each AU, OpenFace yields a variable indicating the presence of an AU in the respective video frame (0—not present, 1—present; denoted by the suffix ‘_c’) as well as an intensity variable providing a continuous output between 1 and 5 (denoted by the suffix ‘_r’). Specifically, the E-DAIC dataset includes the following AUs: AU1, AU2, AU4, AU5, AU6, AU7, AU9, AU10, AU12, AU14, AU15, AU17, AU20, AU23, AU25, AU26, AU28, and AU45. For more information regarding the description of each AU, refer to refs. ^59,60^.

**Fig. 4:**
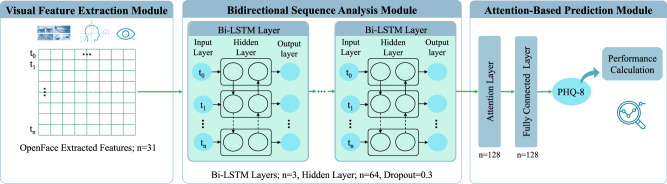
Overview of the proposed framework for depression detection using visual data. (1) Visual feature extraction module using OpenFace^42^. (2) Bidirectional sequence analysis module with Bi-LSTM layers. (3) Attention-based prediction module for PHQ-8 score estimation.

The proposed architecture for predicting PHQ-8 scores from video data is depicted in Fig. 4. The E-DAIC dataset provides pre-extracted features from OpenFace^42^, including head pose, eye gaze, and AUs for each video frame, totaling *n* = 49 features (6 head pose features, 8 eye gaze features, 17 AU intensities, and 18 AU occurrences). These pre-extracted features serve as inputs to the model. As shown in Fig. 4, the model leverages a bidirectional LSTM network architecture^61^ to capture temporal dependencies in sequential data^62^. The architecture consists of three layers of bidirectional LSTM units with 64 hidden units each. Additionally, OpenFace outputs an extraction confidence score per video frame, ranging from 0 to 1. To mitigate the impact of noisy or incomplete data on the model performance, we excluded video frames with a confidence score below 0.90 from further analysis. To accommodate variable-length video frames, we applied padding with zero to equalize all frames to the same length. To prevent overfitting during training, we applied a dropout rate of 0.3 as a regularization technique. Furthermore, we incorporated an attention mechanism consisting of a single attention layer to dynamically weigh the importance of each input feature based on its relevance to the prediction task.

We trained the proposed LSTM model exclusively on the train set, exploring various feature combinations as input, such as head pose features, eye gaze features, AU intensities, and AU occurrences. This also included combinations of two, three, or all feature types, such as AU intensities with head pose and eye gaze. Using the Adam optimizer with a learning rate of 0.1, the model was trained over 20 epochs to minimize mean squared error (MSE) loss. For evaluation, the development and test sets were left untouched, ensuring that performance assessments via root mean square error (RMSE) and MAE metrics allow for direct comparability with existing studies employing the same dataset configuration.

### Multimodal features for automated depression assessment

**Fig. 5:**
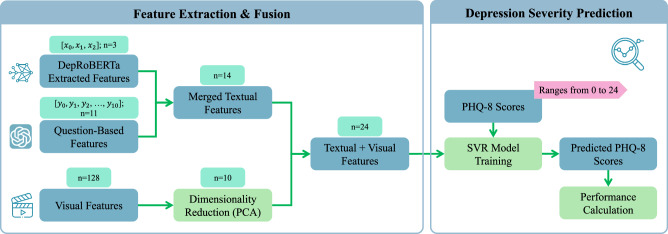
Overview of the multimodal depression detection framework. (1) Feature extraction and fusion, combining 3 DepRoBERTa^49^ textual features, 11 question-based features, and 128 visual features, followed by PCA and fusion into a 24-dimensional representation. (2) Depression severity prediction using an SVR model to estimate PHQ-8 scores with performance evaluation.

To harness the complementary strengths of visual and textual features, we conducted a third experiment in which we combined our two previous prediction pipelines (Sections “Dataset description” and “Textual features for automated depression assessments”). Specifically, we fused the outputs of the DepRoBERTa model, the features extracted by the LLM-driven question-based method, and the visual features extracted by the LSTM model, thereby creating a unified feature space that captures both nonverbal behavioral patterns and linguistic cues potentially indicative of depression. The proposed multimodal framework is depicted in Fig. 5. Among the LSTM models trained on different combinations of visual features as mentioned in the previous section, we identified the optimal model for feature extraction. This model was then used to extract 128-dimensional representations for each data sample. To reduce the dimensionality of these representations, we applied principal component analysis (PCA) with a fixed number of 10 output components. The resulting PCA-transformed LSTM features were then merged with the textual features. Following dimensionality reduction, we employed a feature selection technique using SelectKBest from the Scikit-learn library^51^ with the F-regression score function to identify the top 10 features most strongly associated with PHQ-8 scores. The combined feature set, paired with PHQ-8 scores as the target variable, served as input to train an SVR model with a radial basis function (RBF) kernel. To optimize the SVR hyperparameters, we utilized GridSearchCV^51^ with fivefold cross-validation, using only the training set while reserving the development and test sets for final evaluation. This approach allowed us to keep the development and test sets untouched, ensuring an unbiased evaluation of our model’s performance and facilitating a direct comparison with other studies. Specifically, we explored the following parameters:

- Regularization parameter (*C*): 0.1, 1, 10, and 100
- Kernel coefficient (*gamma*): ‘scale’, ‘auto’, 0.1, 1, and 10
- Epsilon parameter (*epsilon*): 0.1, 0.2, 0.5, and 1

Finally, we evaluated the performance of the SVR model using RMSE and MAE metrics on both the development and test sets.

Moreover, to assess the resilience and adaptability of our multimodal model, we performed a nested cross-validation analysis on the merged train and development sets. This approach involved an external fivefold cross-validation loop for assessment, paired with an internal fivefold loop focused on hyperparameter optimization. Inside the internal loop, we leveraged GridSearchCV^51^ to fine-tune key hyperparameters, including the regularization parameter, kernel coefficient, and epsilon for the SVR model. Furthermore, we applied PCA and SelectKBest to streamline the feature set prior to training.

## Results and discussion

### Outcomes of depression evaluation using textual data

The prediction results of our different approaches for depression assessment based on the E-DAIC interview transcripts are listed in Table 2. To ensure comparability with existing methods, we included results from previous studies using the DAIC and E-DAIC datasets, many of which participated in the 2016, 2017, or 2019 AVEC challenges^27^. We focused on studies that used the PHQ-8 score as the target variable. To highlight one of our main goals—providing a fully automated processing pipeline—we distinguished between fully automated approaches and those requiring manual processing. Therefore, the table’s final column specifies whether each study underwent automated processing of transcripts and extraction of relevant features.

**Table 2:** Performance comparison of PHQ-8 score prediction models using textual features from DAIC (or E-DAIC) dataset

Method	Dataset	MAE (dev^a^)	RMSE (dev)	MAE (test)	RMSE (test)	Auto Proc
Williamson et al.^29^	DAIC	3.34	4.46	–	–	No
Gong et al.^28^	DAIC	**2.77**	**3.54**	3.96	4.99	No
Yang et al.^65,b^	DAIC	3.52	4.52	–	–	No
Stepanov et al.^32^	DAIC	–	–	4.88	5.83	No
Ray et al.^40^	E-DAIC	–	4.37	4.02	4.73	No
Oureshi et al.^77^	DAIC	3.78	–	–	–	No
Niu et al.^78^	DAIC	3.73	4.80	–	–	No
Fang et al.^63^	DAIC	–	–	**3.61**	4.76	No
Rohanian et al.^79^	DAIC	–	–	4.98	6.05	Yes
Makiuchi et al.^37^	E-DAIC	–	–	4.22	6.88	Yes
Al Hanai et al.^31^	DAIC	5.18	6.38	–	–	Yes
Qureshi et al.^69^	DAIC	3.74	4.80	–	–	Yes
Sadeghi et al.^41^	E-DAIC	3.65	5.27	4.26	5.36	Yes
Pr1 + Revised	E-DAIC	4.15	5.28	4.73	6.02	Yes
Pr2 + Revised	E-DAIC	3.87	5.15	4.50	5.61	Yes
Pr3 + Revised	E-DAIC	3.79	4.93	4.36	5.42	Yes
Pr1 + Whisper	E-DAIC	3.99	5.26	4.65	5.95	Yes
Pr2 + Whisper	E-DAIC	3.90	5.10	5.00	6.23	Yes
Pr3 + Whisper	E-DAIC	3.17	4.51	4.22	5.07	Yes
Pr3 + Whisper + AudioQual	E-DAIC	**2.85**	**4.02**	**3.86**	**4.66**	Yes

The best-performing results of this study and previous results that outperformed our results are highlighted in bold. ‘–’ indicates that the respective metrics were not reported in the publication. “Auto Proc” indicates whether each processing step was performed automatically (Yes) or involved manual processing for at least one step (No).

^a^Development.

^b^This study reported separate results for males and females, which we averaged for comparison.

All proposed methods employed transcript transformation using GPT-3.5-Turbo-0125 and a fine-tuned DepRoBERTa model combined with our question-based feature extraction method. We experimented with various prompts (1, 2, and 3) applied to both the original Whisper-generated transcripts and revised transcripts using the Clean-up prompt. Each prompt is described in the Section “Textual features for automated depression assessment”. In the proposed methods Pr1 + Revised, Pr2 + Revised, and Pr3 + Revised, we used revised transcripts and applied prompts 1, 2, and 3, respectively. In the proposed methods Pr1 + Whisper, Pr2 + Whisper, and Pr3 + Whisper, we used the original Whisper-generated transcripts and applied prompts 1, 2, and 3, respectively. Additionally, Pr3 + Whisper + AudioQual integrated speech quality assessment into the Pr3 + Whisper pipeline, analyzing only interviews with acceptable speech quality. This speech quality assessment was performed on all train, development, and test sets, and only interviews of acceptable speech quality were utilized for further analysis.

Our results demonstrated that Pr3 + Whisper achieved the best performance among methods without speech quality assessment, with an MAE of 3.17 and RMSE of 4.51 on the development set, and an MAE of 4.22 and RMSE of 5.07 on the test set. However, the best overall results were obtained using Pr3 + Whisper + AudioQual, with an MAE of 2.85 and RMSE of 4.02 on the development set, and an MAE of 3.86 and RMSE of 4.66 on the test set. As shown in Table 2, Gong et al.^28^ achieved better results on the development set with an MAE of 2.77 and RMSE of 3.54, though their method involved substantial manual processing, including cleaning transcripts, extracting topics, and creating interview questions, which complicates direct comparison. Additionally, Ray et al.^40^ and Williamson et al.^29^ achieved better RMSE on the development set compared to Pr3 + Whisper, however, their approaches also included manual processing at various stages.

On the test set, Gong et al.^28^, Ray et al.^40^, and Fang et al.^63^ reported superior results compared to Pr3 + Whisper, with MAEs of 3.96, 4.02, and 3.61, and RMSEs of 4.99, 4.73, and 4.76, respectively. Ray et al.^40^ manually cleaned the transcripts, although the extent of this cleaning was not fully detailed. Fang et al.^63^ did not specify whether their results were based on the test or development set; we assumed they used the test set. They manually segregated segments where the participant was speaking from the rest of the interview and standardized oral expressions by expanding abbreviations while preserving tone markers such as ‘umm’ or ‘hmm’. Due to these manual processing steps and their use of the DAIC dataset, which differs in participant numbers and PHQ-8 score distribution from the E-DAIC dataset, direct comparisons are challenging.

We selected Pr3 + Whisper as a baseline for integrating speech quality assessment because it achieved the best performance compared to other methods. The resulting model, Pr3 + Whisper + AudioQual, outperformed all previous studies on the test set, including those involving manual transcript processing. Notably, only Gong et al.^28^ surpassed our results on the development set, and Fang et al.^63^ achieved better MAE on the test set. However, both approaches involved manual processing and used the DAIC dataset, making direct comparisons challenging, as mentioned above.

In contrast to previous studies, our approach stands out for its automated pipeline, eliminating the need for manual processing and transcript cleaning. This distinction is crucial, as manual interventions can introduce variability and bias, compromising the model’s generalizability. By leveraging Pr3 + Whisper and integrating speech quality assessment, we achieved superior performance on the test set without relying on manual processing. This automated approach not only streamlines the process but also ensures consistency and reproducibility. Our results demonstrate the importance of high-quality input data as low-quality audio can compromise the model’s performance, leading to inaccurate judgments. Consequently, rigorous quality assessments are essential to ensure reliable predictions, particularly for individuals with high PHQ-8 scores who may otherwise be misclassified as having low scores, resulting in potential missed depression diagnoses.

In an additional experiment where we bypassed the transcript transformation step, the results demonstrated a substantial decline in model performance. When both DepRoBERTa and question-based features were used, we observed an MAE of 4.31 and RMSE of 5.58 on the development set, and an MAE of 4.91 and RMSE of 6.22 on the test set. Without the question-based features, the performance worsened further, with an MAE of 4.69 and RMSE of 5.96 on the development set, and an MAE of 5.55 and RMSE of 7.11 on the test set. In contrast, our best-performing model without speech quality assessment (Pr3 + Whisper with transcript transformation) achieved significantly lower error rates. These results indicate that transcript transformation using the GPT model significantly enhances the extraction of depression-related features, thereby improving the DepRoBERTa model’s accuracy. The marked decline in performance when using raw transcripts highlights the crucial role of this transformation step in optimizing feature extraction and achieving higher prediction accuracy.

To further assess the robustness of our best-performing model without speech quality assessment, we applied the nested cross-validation procedure described in Section “Methods” to the Pr3 + Whisper method. This analysis combined the train and development sets (219 samples) while leaving the test set untouched for final evaluation. The nested cross-validation yielded a mean MAE of 3.39 and a mean RMSE of 4.50 on the development set. The final model selected through this process (*C* = 10, *epsilon* = 0.1, *gamma* = 0.1, *kernel* = RBF) achieved an MAE of 4.52 and an RMSE of 5.47 on the test set. In comparison, the original evaluation of the Pr3 + Whisper method without nested cross-validation showed better performance on the test set. This indicates that while nested cross-validation provided a more rigorous approach with additional hyperparameter tuning, it did not necessarily enhance the model’s generalization on unseen test data. The original Pr3 + Whisper approach appeared to maintain a better balance between the development and test set performance. Furthermore, it is worth noting that the results from the nested cross-validation analysis cannot be directly compared with other studies, as those studies exclusively trained on the train set and evaluated on untouched development and test sets, which is more aligned with the original Pr3 + Whisper evaluation strategy.

### Outcomes of depression evaluation using visual data

Table 3 presents a comparison of our proposed method with previous studies that utilized visual features from the DAIC or E-DAIC datasets to predict PHQ-8 scores. Notably, we achieved the best results regarding the MAE on the test set by combining AU intensities, head pose, and eye gaze features, which are reported as LSTM-AU+pose+gaze in the table. This combination includes a total of 31 features (6 head pose features, 8 eye gaze features, and 17 AU intensities), resulting in an MAE of 4.22 and RMSE of 4.98, outperforming other feature combinations, such as using only AU intensities. Although Fang et al.^63^ reported a lower MAE of 4.12, as mentioned earlier, a direct comparison is challenging due to differences in datasets and evaluation sets. On the development set, our study yielded an MAE of 4.74 and an RMSE of 5.66. Notable exceptions to our results are several studies that achieved better scores. Yang et al. (2016) achieved an MAE of 3.19 and RMSE of 4.29^64^. Yang et al. obtained an RMSE of 5.40^65^. Additionally, Sun et al.^66^, Song et al.^67^, and Du et al.^68^ achieved MAEs of 4.60, 4.37, and 4.61, respectively. However, these studies, which utilized the DAIC dataset, are not directly comparable to our study due to the differences in datasets.

**Table 3:** Performance comparison of PHQ-8 score prediction models using visual features from DAIC (or E-DAIC) dataset

Method	Dataset	MAE (dev^a^)	RMSE (dev)	MAE (test)	RMSE (test)
Nasir et al.^30^	DAIC	6.48	7.86	–	–
Valstar et al.^80^	DAIC	5.88	7.13	6.12	6.97
Yang et al.^64^^2^	DAIC	**3.19**	**4.29**	–	–
Williamson et al.^29^	DAIC	5.33	6.45	–	–
Yang et al.^65^^, b^	DAIC	4.75	**5.40**	–	–
Sun et al.^66^	DAIC	**4.60**	5.90	–	–
Dang et al.^81^	DAIC	5.33	6.67	–	–
Ringeval et al.^82^	DAIC	–	–	6.12	6.97
Stepanov et al.^32^	DAIC	–	–	5.36	6.72
Song et al.^67^	DAIC	**4.37**	5.84	–	–
Ringeval et al.^27^	E-DAIC	–	7.02	–	10.00
Du et al.^68^	DAIC	**4.61**	5.78	–	–
Makiuchi et al.^37^	E-DAIC	–	5.74	–	–
Ray et al.^40^	E-DAIC	–	5.70	–	–
Qureshi et al.^69^	DAIC	–	–	5.06	6.53
Gupta et al.^83^	DAIC	–	–	5.30	6.26
Fang et al.^63^	DAIC	–	–	**4.12**	5.44
LSTM-AU + pose + gaze	E-DAIC	**4.74**	**5.66**	**4.22**	**4.98**

The best-performing results of this study and previous results that outperformed our results are highlighted in bold. ‘–’ indicates that the respective metrics were not reported in the publication.

^a^Development.

^b^This study reported separate results for males and females, which we averaged for comparison.

### Outcomes of depression evaluation using multimodal data

Table 4 illustrates the results of our multimodal method alongside previous studies that have considered text and video-based features for predicting PHQ-8 scores on the DAIC or E-DAIC datasets. As shown in the table, we conducted two analyses. The first analysis is based on the best-performing text-based model without incorporating speech quality assessment. The second analysis includes speech quality assessment and is based on the most successful model in this regard. We refer to these methods as LSTM-AU + pose + gaze + Pr3 + Whisper and LSTM-AU + pose + gaze + Pr3 + Whisper + AudioQual, respectively. Using the LSTM-AU + pose + gaze + Pr3 + Whisper method, we achieved an MAE of 3.31 and an RMSE of 4.65 on the development set, and an MAE of 4.16 and an RMSE of 4.99 on the test set. With the LSTM-AU + pose + gaze + Pr3 + Whisper + AudioQual method, the MAE was 3.01 on the development set and 3.76 on the test set, while the RMSE was 4.18 on the development set and 4.53 on the test set. These results outperform our video-only approach (LSTM-AU + pose + gaze) on both the development and test sets. However, compared to the text-based methods, the multimodal models show worse performance on the development set but achieve slightly better error metrics on the test set. Notably, we compare the LSTM-AU + pose + gaze + Pr3 + Whisper model with the Pr3 + Whisper method and the LSTM-AU + pose + gaze + Pr3 + Whisper + AudioQual model with the Pr3 + Whisper + AudioQual method. This ensures a fair comparison, as the text-based components are consistent within each pair of methods.

**Table 4:** Performance comparison of PHQ-8 score prediction models using textual and visual features from the DAIC (or E-DAIC) dataset

Method	Dataset	MAE (dev^a^)	RMSE (dev)	MAE (test)	RMSE (test)
Ray et al.^40^	E-DAIC	–	4.64	–	–
Qureshi et al.^69^	DAIC	–	–	**3.65**	5.11
Fang et al.^63^	DAIC	–	–	**3.36**	**4.48**
LSTM-AU+pose+gaze+					
Pr3+Whisper	E-DAIC	3.31	4.65	4.16	4.99
LSTM-AU+pose+gaze+					
Pr3+Whisper+AudioQual	E-DAIC	**3.01**	**4.18**	**3.76**	**4.53**

The best-performing results of this study and previous results that outperformed our results are highlighted in bold. ‘–’ indicates that the respective metrics were not reported in the publication.

^a^Development.

The exploration of both video and text modalities simultaneously has been relatively limited in previous studies. As shown in Table 4, Ray et al.^40^ only reported an RMSE of 4.64 on the development set of the E-DAIC dataset without providing additional error metrics. In addition, Qureshi et al.^69^ achieved an MAE of 3.65 and RMSE of 5.11 on the test set of the DAIC dataset, while Fang et al.^63^ reported an MAE of 3.36 and RMSE of 4.48. However, due to the variability in datasets, comparison with these two mentioned studies becomes challenging. Similarly, Qureshi et al.^69^ also observed that integrating text and video modalities did not necessarily lead to the best results and found that relying solely on the text modality yielded superior results.

In an effort to rigorously validate the robustness of our best-performing model, excluding speech quality assessment, we conducted nested cross-validation on the LSTM-AU+pose+gaze+Pr3+Whisper approach. The nested cross-validation process resulted in a mean MAE of 3.42 and a mean RMSE of 4.54 on the development set. The final model selected from this procedure (*C* = 10, *epsilon* = 1, *gamma* = 0.1, *kernel* = RBF) achieved an MAE of 4.22 and an RMSE of 5.08 on the test set. Notably, the original evaluation of the LSTM-AU + pose + gaze + Pr3 + Whisper method without nested cross-validation demonstrated superior performance on the test set. This difference suggests that while nested cross-validation provides thorough hyperparameter optimization, it may lead to slight overfitting, reducing generalization to the test set. The original model, with a consistent train-test split, likely better captured the dataset’s structure, leading to more stable test performance.

**Fig. 6:**
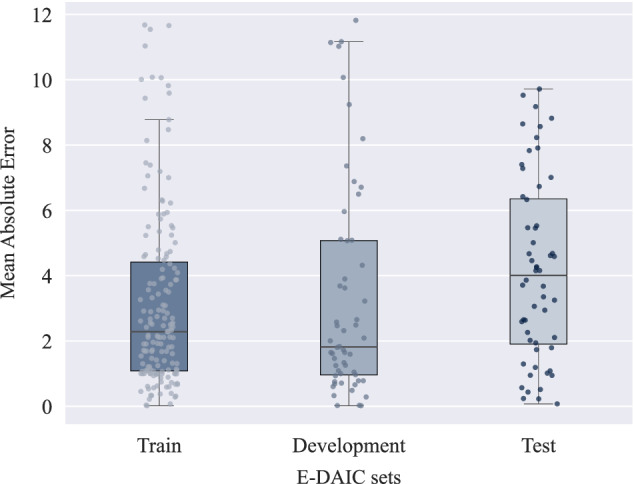
Mean absolute error (MAE) score distribution in the train, development, and test sets for the proposed multimodal approach. The box plots display the median (horizontal line within each box), the interquartile range (IQR; the bounds of the box), and whiskers extending to 1.5× IQR from the box edges. Each point represents the MAE for an individual sample, providing a detailed view of the sample-level variability within each dataset. The *y*-axis reflects the MAE values, where lower scores indicate better performance.

To further illustrate the performance of our multimodal approach, Fig. 6 presents the distribution of MAE scores across the train, development, and test sets using the LSTM-AU + pose + gaze + Pr3 + Whisper + AudioQual method. The plot reveals that the median MAE is highest for the test set, followed by the train set, and then the development set. This indicates that while the model generalizes reasonably well, it faces slightly greater challenges when applied to the test set. The train set exhibits a narrower range of MAE scores, indicating more stable performance during training. In contrast, the wider distributions in the development and test sets suggest that the model experiences greater variability in its predictions on new data. Since errors in the train set are not substantially lower than in the other sets, it indicates that the model is not overfitting. This balance suggests that the model maintains good generalization without being overly optimized for the training data. Additionally, the broader range and presence of outliers in the development and test sets imply that certain data points are more challenging for the model to predict accurately. A major factor contributing to this variability is the distribution of PHQ-8 scores within the dataset, as shown in Fig. 3. High PHQ-8 scores are relatively rare, leading to an imbalance across the sets. This scarcity of samples with severe depression scores makes it more challenging for the model to predict higher PHQ-8 scores accurately, thereby increasing error variability. Differences in input feature quality and the inherent complexity of certain samples contribute to prediction errors. Additionally, the scarcity of high-score instances hinders the model’s ability to generalize, highlighting the need for strategies to address data imbalances.

**Fig. 7:**
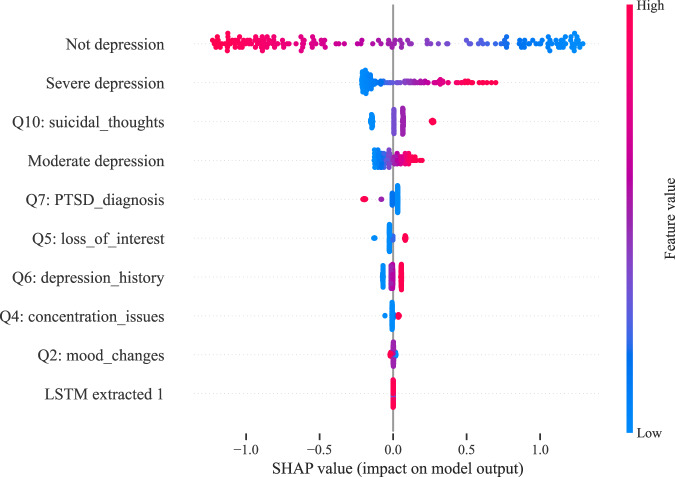
SHAP analysis of the top 10 features in the multimodal approach. ‘Q’ labels indicate questions extracted via the LLM-driven method. ‘Not depression,’ ‘Moderate depression,’ and ‘Severe depression’ are DepRoBERTa-derived indicators. ‘LSTM extracted 1’ is the first component of a 128-dimensional feature vector from the visual modality using the LSTM model.

After assessing the model’s performance across different sets, it was also essential to identify which features had the most influence on the predictions. To this end, we performed a SHapley Additive exPlanations (SHAP)^70^ analysis to highlight the most impactful features. As shown in Fig. 7, this analysis illustrates the relative importance of both text-based and visual features in predicting PHQ-8 scores, focusing on the top 10 selected features for the multimodal model. The results indicate that the most influential features are ‘Not depression’ and ‘Severe depression,’ both extracted by the DepRoBERTa model. As seen in the plot, higher values of the ‘Not depression’ feature are associated with lower predicted PHQ-8 scores, whereas higher values of the ‘Severe depression’ and ‘Moderate depression’ features result in higher predicted scores. This suggests that the model correctly interprets stronger indicators of depression as leading to higher severity scores. Among the text-based features derived from our LLM-driven question-based method, the feature ‘Q10,’ related to the question on suicidal thoughts, is particularly impactful. The SHAP analysis shows that when the value of this feature increases, the predicted PHQ-8 score also rises, indicating its strong association with higher depression severity. Additionally, the features labeled ‘Q1,’ ‘Q2,’ and so on, correspond to the responses to each respective question in our feature extraction process, with ‘Q1’ derived from the first question, ‘Q2’ from the second, and so forth. Notably, the only feature extracted from the visual modality (‘LSTM extracted 1’) appears at the bottom of the list, suggesting that visual features have a much lower impact on the model’s predictions compared to textual features. This observation aligns with the earlier feature selection process, where 9 out of the top 10 features were text-based, with only one derived from visual data. These findings underscore that, within our multimodal framework, text-based features are far more influential in predicting depression severity, while visual features contribute to a lesser extent.

### Toward more effective multimodal depression detection: limitations, insights, and future directions

As discussed in the previous section, our multimodal models demonstrate better performance on the test set compared to text-only approaches, while text-only models perform better on the development set. Additionally, the multimodal models outperform the video-only approach on both the development and test sets. To better understand this pattern, it is insightful to examine the performance of the video-only approach. As shown in Table 3, the video-only model achieves better error metrics on the test set compared to the development set. In contrast, Table 2 reveals that for all text-based models, the error metrics are consistently better on the development set than on the test set. This suggests that integrating text and video enables the multimodal model to achieve better performance on the test set than the text-only model, although the improvements are marginal. One possible explanation for the performance difference between the development and test sets across different data types may be related to the collection process of the E-DAIC dataset. The test set consists exclusively of interviews conducted by an autonomous AI interviewer, whereas the development set includes a mix of interviews controlled by both a human (Wizard-of-Oz) and the AI^25^. This distinction could introduce a distribution shift, affecting how the models perform across the two sets. The presence of a human interviewer in the development set may result in richer and more engaging interview transcripts, thereby enabling text-based models to perform better on the development set. Conversely, the autonomous AI in the test set might lead to less expressive or less detailed responses, diminishing the effectiveness of text-based features.

Despite the multimodal approach showing better performance on the test set compared to text-only models, the improvements remain modest. Feature importance analysis highlights that text data is a crucial component in the model’s predictive power. Text data, especially from sources like social media posts, therapy transcripts, and personal journals, often contains explicit and detailed information about emotional states and thought processes. Symptoms of depression, such as hopelessness, worthlessness, and self-deprecating thoughts, are often directly articulated in language, providing clear indicators for detection models^71–73^. However, integrating text and video data in a multimodal approach introduces additional complexities. Aligning and combining information from different modalities requires sophisticated techniques for temporal synchronization, feature scaling, and data fusion, which may not always be optimal. This integration can introduce noise and redundancy, where conflicting information from one modality can adversely affect overall model performance. These challenges help explain why text-based models often outperform video-based and multimodal models in our study. In contrast, video data presents additional challenges^74^. Non-verbal cues, such as facial expressions and body language, may vary greatly among individuals and situations, making them difficult to interpret accurately. Moreover, facial expressions might not always reflect true emotional states; for instance, someone could smile while discussing distressing experiences. Extracting meaningful features from video involves complex tasks such as facial expression analysis, gesture recognition, and emotional state detection, which can be error-prone due to variations in lighting, camera angles, and individual differences in expressiveness^74,75^. Additionally, the robustness of video-based models can be compromised by the noisy and variable nature of video data, which may contain irrelevant or redundant information. The strengths of LLMs lie in their ability to identify the most relevant parts of interview transcripts related to depression or mental health. However, such methods have yet to be effectively applied to video data. One suggestion for future work is to leverage state-of-the-art LLMs to first identify the most critical segments of an interview related to depression from text data. Subsequently, these key segments could be mapped to the corresponding video frames, allowing the analysis to focus only on specific video portions. This targeted approach could reduce noise and improve the effectiveness of multimodal models.

In addition to these challenges, a notable limitation of the E-DAIC dataset is the relatively small number of samples with high PHQ-8 scores. This means that when the model is trained on such limited high-score samples, its performance on the test set, especially with high PHQ-8 score samples not sufficiently represented during training, can be suboptimal. To maintain comparability with previous studies, we intentionally did not alter the dataset structure using techniques like oversampling or undersampling. Future research could benefit from collecting more balanced datasets that include a representative number of high PHQ-8 score samples, allowing models to be trained and evaluated more effectively across all levels of depression severity. Such balanced datasets could help improve model performance and generalizability by providing a clearer understanding of varying depressive symptoms. To address these limitations, we are conducting a randomized-controlled trial^76^ within the Collaborative Research Center (CRC 1483) “EmpkinS” (Empatho-Kinesthetic Sensor Technology—Sensor Techniques and Data Analysis Methods for Empatho-Kinesthetic Modeling and Condition Monitoring). This trial aims to establish a comprehensive dataset with balanced samples representing various levels of depressive symptoms: none, mild, moderate, and severe. The dataset will comprise extensive video, audio, and biosignal recordings, including electromyography (EMG) to measure muscle activity, electrocardiography (ECG) to monitor heart activity, and respiratory signals (RSP) to track breathing patterns. Our objective is to analyze these multimodal data streams to better understand the links between body language, physical behavior, and depressive symptoms. The insights gained from this research could contribute to the development of more accurate depression detection models, ultimately supporting more effective and personalized mental health interventions.

Beyond addressing the technical challenges and opportunities in multimodal depression detection, it is vital to consider the broader implications of integrating AI technologies into healthcare. Our study illustrates the potential of AI tools in detecting depression through a publicly available, anonymized dataset. The findings emphasize the capability of LLMs and visual cues in identifying depressive symptoms. However, it is important to recognize the limitations of these tools and approach their integration into clinical practice carefully. While AI can enhance screening processes and support healthcare professionals, it is not meant to replace human judgment. Thus, considering the ethical implications and potential biases of incorporating AI technology into healthcare is essential.

## Supplementary information

Supplementary information

## Data Availability

The dataset used in this study is available upon request at https://dcapswoz.ict.usc.edu/.
