# Supplementary material for: Harnessing multimodal approaches for depression detection using large language models and facial expressions
Source: Npj Ment Health Res. 2024 Dec 23;3:66. doi: 10.1038/s44184-024-00112-8 (PMC11666580; doi:10.1038/s44184-024-00112-8)
Supplement: Supplementary file 1 — Supplementary information [file 44184_2024_112_MOESM1_ESM.pdf]

Sample Outputs from LLM Prompts and Fine-Tuned DepRoBERTa Model

In this section, we present sample outputs generated by the GPT-3.5-Turbo-0125 model for transcript transformation using Prompts 1, 2, and 3, as well as the features extracted by the fine-tuned DepRoBERTa model. Additionally, we include the responses from the LLM-driven question-based feature extraction method (Prompt 4). Below, we provide examples for a participant with a PHQ-8 score of 13 from the test set of the E-DAIC dataset. For clarity, the prompts are restated here:

- Prompt 1: “Your task is to read the following text which is an interview with a person and to summarize the key points that might be related to the depression of the person.”
- Prompt 2: “Your task is to read the following text which is an interview with a person and to summarize the key points that might be related to the depression of the person. Please be concise and write your response from the first-person perspective, as if you are the interviewee narrating about your own experiences.”
- Prompt 3: “Could you provide a summary of the main points concerning the mental health of the interviewee from the interview?”
- Prompt 4 (LLM-driven Question-based Method): “Can you answer these questions from this text which is an interview with a person only with ‘YES’ or ‘NO’ or ‘To Some Extent’? If the question or corresponding answer is not found, answer ’Not Mentioned’. These are the questions: (1) Have you felt emotionally and physically well lately?; (2) Have you noticed significant changes in your mood, such as feeling persistently sad, empty, or hopeless?; (3) Have you experienced difficulties with your sleep, such as trouble falling asleep, staying asleep, or waking up too early?; (4) Are you finding it challenging to concentrate on tasks or make decisions?; (5) Have you lost interest or pleasure in activities you used to enjoy?; (6) Have you ever been diagnosed with depression or experienced prolonged periods of feeling down or hopeless in the past?; (7) Have you ever been diagnosed with PTSD (Post- Traumatic Stress Disorder)?; (8) Have you been experiencing any financial problems recently?; (9) Do you find it challenging to socialize and prefer solitary activities, indicating introverted tendencies; (10) Have you had thoughts of death or suicide, or have you made any suicide attempts?; (11) Have you ever served in the military?”

- **Responses from Prompt 1**
  - “Difficulty sleeping and being jolted awake, which are symptoms of PTSD”
  - “Trouble with trust and feeling worthless after being pushed off a tower during training”
  - “Difficulty coping with triggers, such as heights and past traumatic events
  - “Regular therapy sessions to cope with PTSD and related symptoms”
  - “Periods of feeling healthy and making progress, but also experiencing spurts of difficulty”
  - “Trying to move forward and finding enjoyment in activities like antique shopping”
  - “Positive influence from the Women’s Health Center and the psychologist there”
  - “Feeling proud of trying to go forward despite challenges and past traumas”

For this transformed transcript, the fine-tuned DepRoBERTa model produced the following probabilities for ‘severe’ depression: 0.646, for ‘moderate’: 0.305, and ‘not depression’: 0.050.

- **Responses from Prompt 2**

"I have been diagnosed with PTSD for seven years now, and I struggle with trouble sleeping and feelings of mistrust. The trigger for my symptoms was related to my airborne training in the military. I have been coping with counseling and trying to overcome my fear of heights. Therapy has been useful for me in dealing with these challenges, and I have noticed some positive changes since I started therapy. Despite the difficulties I face, I am proud of my efforts to move forward and seek help."

In this case, the DepRoBERTa model assigned probabilities of 0.911 for 'severe' depression, 0.078 for 'moderate', and 0.012 for 'not depression'.

- **Responses from Prompt 3**

"The interviewee discussed various aspects of their mental health throughout the inter- view. They mentioned experiencing trouble sleeping, being triggered by past traumatic experiences, being diagnosed with PTSD, and attending therapy to cope with their challenges. The interviewee also shared their struggles with anxiety, fear, and feelings of worthlessness related to their military training. Despite these difficulties, therapy was described as helpful in managing their symptoms and making progress towards feeling healthier. The Women's Health Center and antique shopping were mentioned as positive influences and enjoyable activities in the interviewee's life. Overall, the interviewee expressed a desire to move forward and improve their mental well-being."

For this prompt, DepRoBERTa assigned probabilities of 0.716 for 'severe' depression, 0.234 for 'moderate', and 0.050 for 'not depression'.

- **Responses from Prompt 4 (Question-based Responses)**

"(1) YES, (2) YES, (3) YES, (4) YES, (5) YES, (6) YES, (7) YES, (8) YES, (9) To Some Extent, (10) YES, (11) YES."

Finally, using the features extracted by DepRoBERTa along with the question- based responses, the SVR model predicted a PHQ-8 score of 12.32 for this participant. These results highlight the strengths and nuances of each prompt and the DepRoBERTa model. Prompt 1 focused on extracting specific symptoms and coping mechanisms, while Prompt 2, by adopting a first-person perspective, captured a more personal and introspective narrative, which may explain why the DepRoBERTa model assigned a higher probability for severe depression. Prompt 3 provided a comprehensive overview, identifying both challenges and positive influences, resulting in a more balanced assessment of moderate depression. The fine-tuned DepRoBERTa model performed consistently well across all prompts, effectively distinguishing between 'severe', 'moderate', and 'no depression' with varying degrees of confidence. Furthermore, the question-based responses from Prompt 4 demonstrated that the participant experienced several core symptoms of depression, aligning with the PHQ-8 score prediction. The SVR model's predicted score of 12.32 closely matched the actual score.
